# Epidemiology and Genetic Characterization of Classical Human Astrovirus Infection in Shanghai, 2015–2016

**DOI:** 10.3389/fmicb.2020.570541

**Published:** 2020-09-25

**Authors:** Limeng Wu, Zheng Teng, Qingneng Lin, Jing Liu, Huanyu Wu, Xiaozhou Kuang, Xiaoqing Cui, Wei Wang, Xiaoxian Cui, Zheng-an Yuan, Xi Zhang, Youhua Xie

**Affiliations:** ^1^Key Laboratory of Medical Molecular Virology (MOE/NHC/CAMS), Department of Medical Microbiology and Parasitology, School of Basic Medical Sciences, Shanghai Medical College, Fudan University, Shanghai, China; ^2^Shanghai Municipal Center for Disease Control and Prevention, Shanghai, China; ^3^Shanghai Institute of Preventive Medicine, Shanghai, China

**Keywords:** viral diarrhea, human astrovirus, epidemiological characteristics, viral load, coinfection

## Abstract

**Objective:**

Human astroviruses (HAstVs) are single-stranded RNA viruses of the *Astroviridae* family. Infection of classic HAstVs is one of the most common causes of acute viral gastroenteritis (infectious viral diarrhea). There is a lack of data on the prevalence and genetic characterization of classic HAstVs in acute viral gastroenteritis in the whole population. This study aimed to investigate the epidemiological trend, genotypes, viral co-infections, and viral loads of classic HAstVs in Shanghai, China, from January 2015 to December 2016.

**Methods:**

A total of 6,051 non-redundant stool samples were collected in outpatients with acute diarrhea in Shanghai from January 2015 to December 2016. One-step real-time RT-PCR was used for screening viral diarrhea, including rotavirus A, rotavirus B, rotavirus C, norovirus genotype I and II, classic human astroviruses, and sapovirus. Real-time PCR was used for screening human enteric adenoviruses. Conventional RT-PCR was used for the amplification of viral fragments for genotyping. PCR products were sequenced and used for the construction of phylogenetic trees.

**Results:**

The detection rate of classic HAstVs was 1.55% (94/6,051). The prevalence of HAstV infection displayed a typical winter/spring (December to March) seasonality and was highest in the 5–14 age group. Eighty-six samples were genotyped, which revealed HAstV-1 as the most prevalent genotype, followed by HAstV-5, HAstV-4, HAstV- 2, HAstV-8, and HAstV-3. There was a dramatic rise in the prevalence of HAstV-4 from December 2015 to March 2016, and the viral loads of HAstV-4 were significantly higher than those of other genotypes. Among the mixed infection samples, noroviruses were found to be the most frequently co-infected enteric viruses with HAstV.

**Conclusion:**

Multiple genotypes of classic HAstVs circulated in Shanghai from January 2015 to December 2016. For the first time, HAstV-3\4\5\8 were detected in Shanghai.

## Introduction

Human astroviruses (HAstVs) are small, non-enveloped, positive-sense single-stranded RNA viruses of the *Astroviridae* family. Classic HAstVs are among the most important viruses causing acute viral gastroenteritis worldwide (Banyai et al., 2018). Infection of classic HAstVs usually causes mild symptoms and has been implicated in gastroenteritis outbreaks indoors, such as in daycare centers and schools (Pankovics et al., 2011; Tan et al., 2019). Mixed infections of HAstVs with rotavirus, norovirus, and adenovirus are common (Chhabra et al., 2013; Li et al., 2016; Liu et al., 2016; Andersson et al., 2018; Pijnacker et al., 2019). In susceptible individuals, including pediatric patients, older adults, and the immunocompromised hosts, HAstVs, especially non-classic human astroviruses (HAstV-VA/HMO and HAstV-MLB), may infect the central nervous system with severe clinical consequences (Fremond et al., 2015; Cordey et al., 2016; Lum et al., 2016; Sato et al., 2016).

The genome of HAstVs harbors three open reading frames (ORFs), namely, ORF1a, ORF1b, and ORF2. ORF1a and ORF1b encode non-structural proteins (nsPs) involved in RNA transcription and replication, while ORF2 encodes the capsid proteins. According to the International Committee on Taxonomy of Viruses (ICTV), HAstVs are classified into three divergent groups, namely, classic HAstVs (mammalian astrovirus 1, MAstV 1), HAstV-MLB (MAstV 6), and HAstV-VA/HMO (MAstV 8 and MAstV 9) (Bosch et al., 2014).

Classic HAstVs segregate into eight genotypes (HAstV-1–HAstV-8), and each corresponds to an individual serotype. Lineages or subtypes have been identified in HAstV-1–HAstV-6, and there are a total of 20 lineages (HAstV-1a to 1f, HAstV-2a to 2d, HAstV-3a and 3b, HAstV-4a to 4c, HAstV-5a to 5c, and HAstV-6a and 6b) (Martella et al., 2014). A new lineage within HAstV-3 (HAstV-3c) was recently proposed (Medici et al., 2015).

Most studies of viral diarrhea etiology focus on children aged under 5 years, which is a partial representation of the general population (Liu et al., 2016; Platts-Mills et al., 2018; Kotloff et al., 2019). Furthermore, recent epidemiological data on HAstV-induced gastroenteritis in China are limited (Shan et al., 2009). Our study also adds to the limited number of studies investigating outpatient cases of HAstV-induced infectious diarrhea in China.

In 2012, Shanghai Municipal Centers for Disease Control and Prevention (CDC) established the Shanghai diarrhea comprehensive surveillance system (SDCSS) and has been taking on continuous surveillance since then. In this study, we analyzed the available SDCSS data from January 2015 to December 2016 to investigate the epidemiological trend, genotypes, viral co-infections, and viral loads of HAstV-associated gastroenteritis in all age groups in Shanghai. The study revealed that multiple genotypes of classic HAstVs circulated in Shanghai and HAstV-3\4\5\8 were discovered in Shanghai for the first time.

## Materials and Methods

### Patients and Samples

Diarrheal patients were defined as those who visited the enteric disease clinics of sentinel hospitals, with three or more loose or liquid stools per day. HAstV-affected patients were defined as those whose stool samples were positive for HAstV PCR detection at the initial screening, including patients with viral co-infections.

A total of 6,051 non-redundant fecal samples were collected using the probability proportional to size sampling method across 22 sentinel hospitals of SDCSS in outpatients with acute diarrhea in Shanghai from 2015 to 2016. The SDCSS system consists of Shanghai municipal CDC, 16 district CDCs, and 22 sentinel hospitals. Sample information was collected through an online questionnaire, including age, gender, treatment time (time of onset), season, and clinical symptoms. The study protocol was reviewed and approved by the Human Research Ethics Committee of the Shanghai Municipal Centers for Disease Control and Prevention.

### Viral Nucleic Acid Extraction

Viral nucleic acids (RNA, DNA) were extracted from 200 μL of 10% fecal suspension in phosphate-buffered saline. The extraction was done using a MagNA Pure LC Total Nucleic Acid Isolation Kit according to the manufacturer’s instructions (Roche Diagnostics, Mannheim, Germany). Viral nucleic acids were eluted in 50 μL of elution buffer and stored at −80°C immediately.

### Real-Time RT-PCR and Conventional RT-PCR

Classic HAstVs, norovirus genogroup I and II, sapovirus, and rotavirus A, rotavirus B, rotavirus C were detected using real-time reverse transcription PCR (RT-PCR) assays. Enteric adenoviruses were detected using real-time PCR. All assays were performed using the respective commercial kits according to the manufacturer’s instructions (Zhijiang Biotechnology, Shanghai, China). Primer information is listed in Supplementary Data Sheet 1.

Conventional RT-PCR was used for confirmation of classic HAstVs and subtyping. RT-PCR was performed using a SuperScript^TM^ III One-Step RT-PCR System with a Platinum^TM^ Taq DNA Polymerase Kit (Invitrogen, United States). Briefly, 10 μL of extracted viral RNA was added to 40 μL of a reaction mixture containing 25 μL of 2 × Reaction Mix, 1 μL sense primer (10 μM), 1 μL anti-sense primer (10 μM), 1 μL SuperScript^TM^ III RT/Platinum^TM^ Taq Mix, and 12 μL autoclaved distilled water. The mixture was reverse transcribed for 30 min at 55°C and 2 min at 94°C. PCR amplification was performed under the following thermal cycling conditions: initial denaturation at 94°C for 2 min, followed by 40 cycles of amplification with denaturation at 94°C for 15 s, primer annealing at 55°C for 30 s, and an extension reaction at 68°C for 1 min and then a final extension at 68°C for 5 min.

Viral loads of HAsVs in fecal samples were measured using quantitative real-time RT-PCR with primers and probe targeting ORF1b (Zhijiang Biotechnology, Shanghai, China). Briefly, 5 μL of sample RNA was mixed with 20 μL of a reaction buffer containing reaction master mix, sense and anti-sense primers, probe, and nuclease-free water. Real-time RT-PCR was performed under the following thermal cycling conditions on a LightCycler 480 real-time PCR instrument II (Roche Diagnostics, Mannheim, Germany): initial reverse transcription at 45°C for 15 min for the synthesis of cDNA, followed by 45 cycles of amplification with denaturation at 94°C for 15 s, and annealing and extension at 62°C for 60 s. Fluorescence readings were collected and analyzed with LightCycler 480 software (version 1.5.1, Roche Diagnostics). The genome copy numbers of HAstVs were determined based on a standard curve prepared with 10-fold serial dilutions of plasmid DNA provided in the commercial kit (Zhijiang Biotechnology, China).

### Phylogenetic Analysis

The PCR products obtained from viral (HAstVs, norovirus genotype I and II, sapovirus, and enteric adenovirus) positive specimens were subjected to Sanger sequencing (Shenggong, Shanghai, China). The sequences were edited using BioEdit software and compared with reference sequences in the NCBI GenBank database using the BLAST server^1^.

The reference strains from GenBank representing different HAstV genotypes were included in the phylogenetic analysis. All the selected sequences (Supplementary Data Sheet 2) were aligned using MEGA 6.06. Neighbor-joining (NJ) trees were constructed using the Kimura two-parameter method, and the reliability was assessed by bootstrap with 1,000 resampling loops. The sequences of diarrheal viruses described in this study were deposited in the GenBank database with the accession numbers listed in Supplementary Data Sheet 3.

### Statistical Analysis

All calculations were conducted using Microsoft Excel 2013 and SPSS software v19.0 (IBM, United States). We determined whether there were statistically significant differences between the different age groups with detected virus using a χ^2^ test and a Fisher exact with a two-tailed method. Differences between HAstV genotypes and the viral loads in samples were determined using analysis of variance (ANOVA). *P-*values of <0.05 were considered statistically significant.

## Results

### Age and Seasonal Characteristics of HAstV Prevalence

**FIGURE 1:**
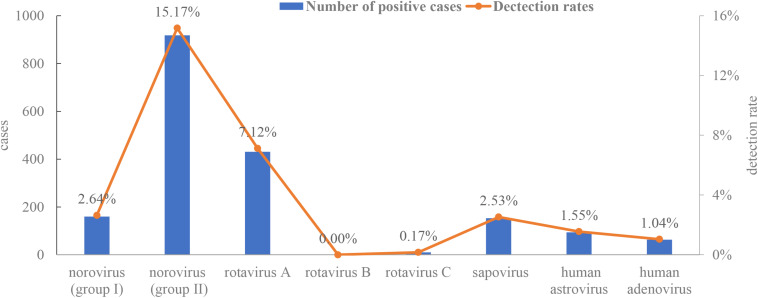
Detection rates of enteric viruses [rotavirus A, rotavirus B, rotavirus C, norovirus (genogroup I and II), adenovirus, human astrovirus, and sapovirus].

A total of 6,051 non-redundant stool samples from diarrheal patients were collected from January 2015 to December 2016. Viral screening using one-step real-time RT-PCR indicated that 28% (1,694/6,051) of the samples contained at least one virus, and 124 samples had two or three viruses. Norovirus genogroup II was the most frequently detected (15.17%), followed by rotavirus A (7.12%), norovirus genogroup I (2.64%), sapovirus (2.53%), HAstVs (1.55%), enteric adenovirus (1.04%), and rotavirus C (0.17%) (Figure 1). No rotavirus B was found in any samples.

**FIGURE 2:**
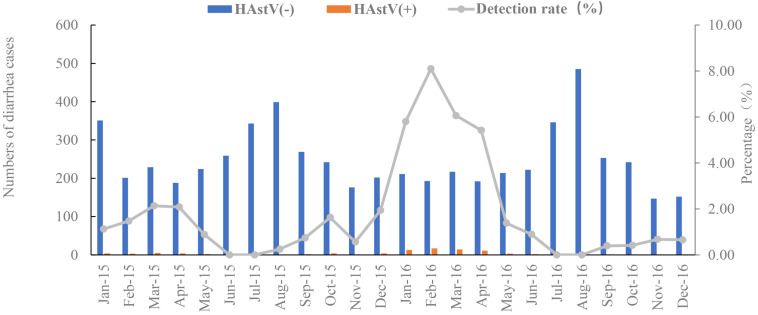
Monthly distribution of HAstV cases and detection rate from January 2015 to December 2016. HAstV(+):patients were positive for classical human astrovirus. HAstV(−):patients were negative for classical human astrovirus. The gray line is the detection rates of HAstV monthly. Blue bar is the negative cases of HAstV infection. Orange bar is the positive cases of HAstV infection.

The 94 HAstV-positive samples were derived from patients across a wide age range from 120 days to 81 years, with a mean age of 32.8 years and a median age of 30 years. The prevalence of HAstVs did not differ significantly between males and females. The highest HAstV positivity rate was in the older children and teenage age group between 5 and 14 years (5.38%, *p* = 0.005) (Table 1) (Supplementary Data Sheet 4). A typical late winter early spring (February to April) seasonality was observed for the prevalence of HAstVs. The prevalence peaked in February (4.83%, *n* = 20) and landed at the lowest level in July (*n* = 0) (Figure 2).

**TABLE 1:** Age distribution of HAstV infections.

	HAstV(+)	HAstV(−)	Detection Rate(%)	*p-*Value
	*n* = 94	*n* = 5957	1.55	
**Gender**				
Male	50	3099	1.59	
female	44	2858	1.52	
**Age(year)**				0.005
0–4	15	893	1.65	
5–14	7	123	5.38	
15–29	25	1201	2.04	
30–44	22	1241	1.74	
45–59	10	1128	0.88	
60–69	8	826	0.96	
70–79	5	354	1.39	
80 +	2	191	1.04	

**HAstV(+):patients were positive for classical human astrovirus. HAstV(−):patients were negative for classical human astrovirus. Significant differences between the different age groups with detected virus using a χ^2^ test and a Fisher exact with a two-tailed method.**

### Genotype and Subgenotype Characteristics of HAstVs

**FIGURE 3:**
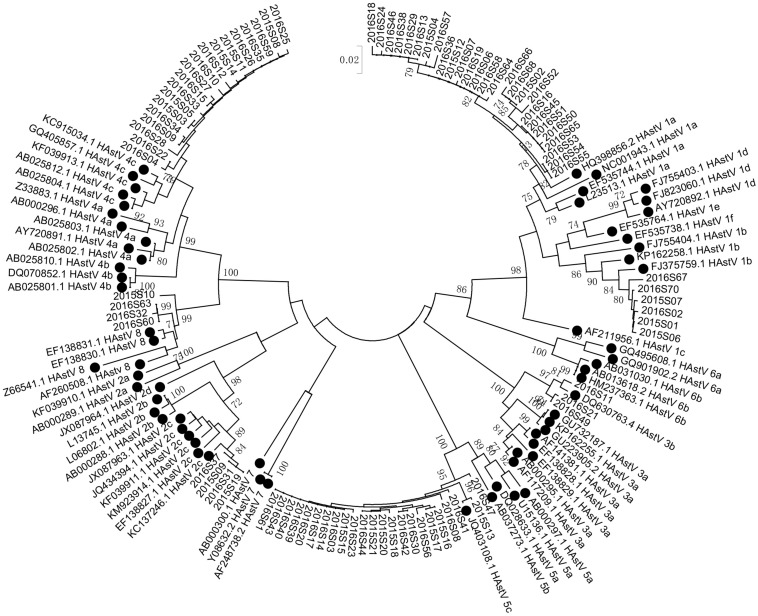
Phylogenetic tree of human astroviruses. Phylogenetic tree of human astroviruses was constructed using a 319 bp alignment of sequences of the ORF2 region by Neighbor-Joining method with 1,000 bootstrap replicates and Tamura 3-parameter model. Reference sequences were denoted with a black cycle. Scale bar indicates nucleotide substitution per site. Bootstrap values (1,000 replicates) above 70 are shown.

**FIGURE 4:**
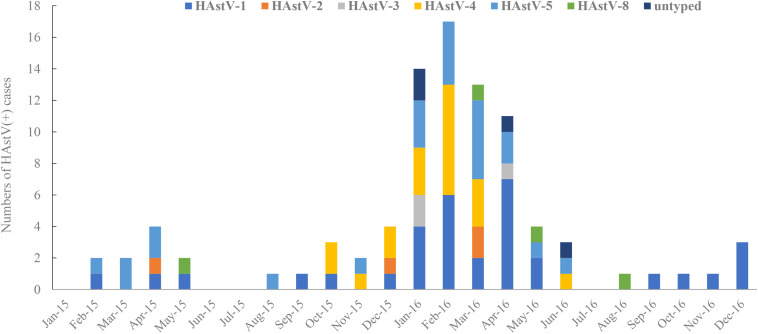
Monthly Distribution of HAstV positive cases and genotypes from January 2015 to December 2016. HAstV-1∼ HAstV-8 represent for classical human astrovirus genotype 1∼8.

HAstV genomic fragments (corresponding to the ORF2 region, approximately 319 nt after removing the primer and unreadable sequences at 3′ and 5′ends) were successfully amplified from 90 of the 94 HAstV-positive samples initially screened. Phylogenetic analysis of the sequences of these fragments revealed six HAstVs genotypes (HAstV-1–5 and HAstV-8) among 86 samples and four non-subtyped samples (Table 2). HAstV-6 and HAstV-7 were not detected. HAstV-1 (36.67%, 33) was the most prevalent genotype, followed by HAstV-5 (25.56%, 23), HAstV-4 (21.11%, 19), HAstV-2 (4.44%, 4), HAstV-8 (4.44%, 4), and HAstV-3 (3.33%, 3). Further subtyping showed that there were 27 HAstV-1a and 6 HAstV-1b. Each of the other HAstVs had only one subtype (HAstV-2c, HAstV-3b, HAstV-4c, and HAstV-5c) (Table 2 and Figure 3). Also, there was a sharp increase in HAstV-4 cases during the winter of 2015 and the spring of 2016 (Figure 4). Using sensitive qRT-PCR, the viral loads in 78 HAstV-positive samples were determined to range from 2.86 × 10^2^ to 7.53 × 10^11^ per gram of stool sample (Supplementary Data Sheet 5). The mean viral load of HAstV-4 was significantly higher than that of other HAstV genotypes (*p* < 0.001). The results indicate that multiple genotypes were circulating in Shanghai from January 2015 to December 2016.

**TABLE 2:** Genotype distribution of HAstV.

	HAstV-1	HAstV-2	HAstV-3	HAstV-4	HAstV-5	HAstV-8	Untyped	Total
*n*	33	4	3	19	23	4	4	90
N%	36.67%	4.44%	3.33%	21.11%	25.56%	4.44%	4.44%	

**HAstV-1∼ HAstV-8 represent for classical human astrovirus genotype 1∼8.**

### Co-infections of HAstV With Other Enteric Viruses

The highest percentage of mixed infection by tested enteric viruses were observed in samples positive for norovirus genogroup I (31.55%, 53/168) and sapovirus (30.63%, 49/160) (Supplementary Data Sheet 6). For HAstV, 28.89% (26/90) of the HAstV-positive samples were found to be positive for at least one other tested virus. Nineteen and seven samples had double (HAstV and one enteric virus) and triple (one HAstV and two enteric viruses) infections (Supplementary Data Sheet 7). The viruses found to most frequently co-infect with HAstV was norovirus (50.0%, 19), followed by sapovirus (28.9%, 11), rotavirus A (13.2%, 5), and enteric adenovirus (7.9%, 3). The noroviruses in 16 of the 19 co-infection samples were genotyped and distributed in eight different genotypes (GI.3, GI.4, GI.5, GI.6, GII.1, GII.4, GII.17, and GII.21). Co-infecting sapoviruses showed a predominance of genogroup I (GI.1, GI.2, GI.3, and GI.6). All the co-infecting rotaviruses were rotavirus A. Adenoviruses in two co-infected samples were genotyped as genotypes 1 and 41 (Supplementary Data Sheet 8).

Among the co-infected cases, HAstV-1 appeared as the most prevalent (7/26), followed by HAstV-5 and HAstV-4 (both were 6/26), HAstV-2, and HAstV-8 (both were 2/26), HAstV-3 (1/26), and two non-genotyped (Table 3), which is the same as the genotype distribution of all HAstV infections.

**TABLE 3:** Correlation between genotypes of HAstV and mixed infection viruses.

	HAstV-1	HAstV-2	HAstV-3	HAstV-4	HAstV-5	HAstV-8	Untyped
Rotavirus	2 (type A)			1 (type A)	1 (type A)		1 (type A)
Norovirus	5 (GI.3,GI.4,GI.6,GII.17)	1 (GII.1)	2 (GI.6,GII.17)	2 (GI.3,GII.17)	6 (GI.5,GII.4,GII.17)		
Sapovirus	1 (GI.1)	2 (GI.2,GI.6)		2 (GI.2,GI.3)		2 (GI.2,GI.6)	1 (GI.2)
Adenovirus				1 (type1)		1 (type41)	
Coinfection Total	8	3	2	6	7	3	2
	33	4	3	19	23	4	4

**HAstV-1∼ HAstV-8 represent for classical human astrovirus genotype 1∼8.**

## Discussion

Acute viral gastroenteritis is one of the most common infectious diseases worldwide. Human astrovirus is currently recognized as a major cause of sporadic gastroenteritis in both children and adults. In China, most published studies have mainly focused on HAstV infections of children, especially children under 5 years old. This study was the first report on the epidemiological trends, genotypes, viral co-infections, and viral loads of HAstV infections of the whole population in Shanghai, based on citywide diarrheal surveillance data from January 2015 to December 2016.

Out of 6,051 stool samples, the HAstV positive rate was 1.55% (including double and triple viral infections). The detection rates of HAstV varied in previous studies on the data from China, ranging from 1.8 to 13.6%, depending on the age group and detecting methods (Dai et al., 2010; Wang et al., 2011, 2013; Ouyang et al., 2012; Lu et al., 2015). Interestingly, the highest detection rate of HAstVs in our study was from the older children and teenage group (5–14 years old) (5.38%, 7/130). The detection rate of HAstVs in the age group under 5 years was 1.65% (15/908), which was similar to a previous report (1.8%, 8/436) (Lu et al., 2015). According to a multisite birth cohort study (MAL-ED) (Platts-Mills et al., 2015), the detection rate of astrovirus was 2.7% in the first year, with an increase to 4.2% in the second year. The increase in the detection rate in the 5–14 years age group might suggest a waning of HAstV antibodies or could be due to more active social interaction. However, compared with other age groups, the 5–14 years age group had the smallest sample size and contained a few cases with close onset dates, which may cause a sampling bias.

Our study showed a typical cold season distribution of HAstV infection with a single peak. The peak from December 2015 to April 2016 was much higher than the previous one. Two peaks of HAstV detection rate were observed in Germany, between February and May and between September and December (Jacobsen et al., 2018). In Korea, the pattern of seasonal distribution varied from one year to another (Kim et al., 2019). The seasonality of HAstV infections in Shanghai needs to be further monitored.

Six genotypes were found to circulate during 2015–2016, with HAstV-1 being predominant, followed by HAstV-5, HAstV-4, HAstV-2, HAstV-8, and HAstV-3. Except for HAstV-1a and 1b, all other HAstVs are of a single subtype/lineage. HAstV-5\4\2\8\3 have not been reported in Shanghai. The detection of these genotypes in this study indicates that multiple HAstVs were circulating in Shanghai. It would be interesting to monitor the frequency of these genotypes in the Shanghai population in the future. Like other recent studies (Jacobsen et al., 2018; Kumthip et al., 2018; Bitencurt et al., 2019; Kim et al., 2019), HAstV-6 and -7 were not detected.

Notably, the peak of HAstV-induced cases in 2016 was almost five times higher than in the preceding year. A rapid rise in HAstV-4 cases was observed during the high HAstV peak season from December 2015 to April 2016. Furthermore, HAstV-4-positive samples showed a high viral load. Multiple factors might have contributed to this result. Compared with the 2015 data, both the case number and detected genotypes of HAstV infections had increased over the same period last year, and HAstV-4 in particular had the most obvious change. The HAstV-4 strains observed in this study may be a variant because they exhibited a characteristically high stool shedding. In addition to clarifying the relationship between this variant and the severity of the disease, it is also necessary to study its infectiousness. As is well known, norovirus infections are a leading cause of acute gastroenteritis worldwide in persons of all age groups. Interestingly, the cases of norovirus infections in January 2016 were almost five times lower than in January 2015 during the same period based on SDCSS data (Gong et al., 2018). In contrast, the incidence of rotavirus infection was similar. There may be some underlying connection between the decrease in the number of norovirus cases and the increase in HAstV cases.

This study has several limitations. First, non-classic HAstVs (VA1, VA2, MLB1, MLB2, VA3, HMO-A-B-C) were not detected. The primers for the initial screening of sentinel hospitals were not suitable for non-classic HAstV detection. Future screening should include the detection of these non-classic HAstVs. Second, no diarrhea-free control group was included in the study. Results from the Hebei Province of China among children showed a high HAstV detection in asymptomatic control children (Shen et al., 2019), suggesting that our data might have over-represented HAstVs’ importance as pathogens. Finally, we did not acquire sufficient clinical information from outpatients. Therefore, the relationship between the gastroenteritis severity and genotype/viral load needs to be further studied.

In conclusion, this is the first report on the epidemiological characteristics of classic human astroviruses in Shanghai from January 2015 to December 2016. Our study pointed out that multiple classic HAstV genotypes circulated simultaneously in Shanghai, and that for the first time, HAstV-3\4\5\8 were detected in Shanghai. There was a sharp increase in HAstV-4 cases during the winter of 2015 and the spring of 2016.

## Data Availability Statement

The datasets presented in this study can be found in online repositories. The names of the repository/repositories and accession number(s) can be found in the article/Supplementary Material.

## Ethics Statement

The studies involving human participants were reviewed and approved by the Shanghai Municipal Center for Disease Control and Prevention Ethical Review Committee (No. 2017-26). Affiliation: Shanghai Municipal Center for Disease Control and Prevention. Written informed consent to participate in this study was provided by the participants’ legal guardian/next of kin.

## Author Contributions

YX and XZ designed this study. LW, QL, JL, HW, and Z-aY analyzed the data. XK and XQC collected the samples. LW, WW, and XXC contributed the samples testing. LW, ZT, QL, XZ, and YX wrote this manuscript. All authors approved the final manuscript as submitted and agreed to be accountable for all aspects of the work.

## Conflict of Interest

The authors declare that the research was conducted in the absence of any commercial or financial relationships that could be construed as a potential conflict of interest.

## Footnotes

1 http://www.ncbi.nlm.nih.gov/blast
