# Supplementary material for: Epidemiology and Genetic Characterization of Classical Human Astrovirus Infection in Shanghai, 2015–2016
Source: Front Microbiol. 2020 Sep 25;11:570541. doi: 10.3389/fmicb.2020.570541 (PMC7546348; doi:10.3389/fmicb.2020.570541)
Supplement: Supplementary file 1 [file Data_Sheet_1.docx]

**Supplementary Data Sheet 1 | The oligonucleotide primers for conventional RT PCR (typing enteric viruses) used in this study.**

| **Virus** | **Primer** | **Polarity** | **Sequence**  **（5’-3’）** | **Ref.** |
| --- | --- | --- | --- | --- |
| Human Astrovirus | Mon269 | Sense | CAACTCAGGAAACAGGGTGT | ^[^[^1^](#_ENREF_1)^]^ |
| (classical) | Mon270 | Antisense | TCAGATGCATTGTCATTGGT |  |
| Norovirus (GI) | G1-SKF | Sense | CTGCCCGAATTYGTAAATGA | ^[^[^2^](#_ENREF_2)^]^ |
|  | Mon432 | Sense | TGGACICGYGGICCYAAY CA |  |
|  | G1-SKR | Antisense | CCAACCCARCCATTRTACA |  |
| Norovirus (GII) | Ring 2 | Sense | TGGGAGGGCGATCGCAATCT | ^[^[^2^](#_ENREF_2)^]^ |
|  | Mon431 | Sense | TGGACIAGRGGICCYAAYCA |  |
|  | G2-SKR | Antisense | CCRCCNGCATRHCCRTTRTACAT |  |
| Sapovirus | SLV-5317 | Sense | CTCGCCACCTACRAWGCBTGGTT | ^[^[^2^](#_ENREF_2)^]^ |
|  | SLV-5749 | Antisense | CGGRCYTCAAAVSTACCBCCCCA |  |
| Adenovirus | Ad1 | Sense | TTCCCCATGGCICAYAACAC | ^[^[^3^](#_ENREF_3)^]^ |
|  | Ad2 | Antisense | CCCTGGTAKCCRATRTTGTA |  |

**Supplementary data sheet 2 |** **Strains used in the study**

| **Genotype (subgenotype)** | **Accession No.** |
| --- | --- |
| Human_astrovirus_2b | AB000288 |
| Human_astrovirus_2a | AB000289 |
| Human_astrovirus_3a | AB000295 |
| Human_astrovirus_4a | AB000296 |
| Human_astrovirus_5a | AB000297 |
| Human_astrovirus_7 | AB000300 |
| Human_astrovirus_6b | AB013618 |
| Human_astrovirus_4b | AB025801 |
| Human_astrovirus_4a | AB025802 |
| Human_astrovirus_4a | AB025803 |
| Human_astrovirus_4c | AB025804 |
| Human_astrovirus_4b | AB025810 |
| Human_astrovirus_4c | AB025812 |
| Human_astrovirus_6b | AB031030 |
| Human_astrovirus_5b | AB037273 |
| Human_astrovirus_3a | AF117209 |
| Human_astrovirus_3a | AF141381 |
| Human_astrovirus_7 | AF248738 |
| Human_astrovirus_8 | AF260508 |
| Human_astrovirus_4a | AY720891 |
| Human_astrovirus_1d | AY720892 |
| Human_astrovirus_5a | DQ028633 |
| Human_astrovirus_4b | DQ070852 |
| Human_astrovirus_3b | DQ630763 |
| Human_astrovirus_2c | EF138827 |
| Human_astrovirus_3a | EF138828 |
| Human_astrovirus_3a | EF138829 |
| Human_astrovirus_8 | EF138830 |
| Human_astrovirus_8 | EF138831 |
| Human_astrovirus_1f | EF535738 |
| Human_astrovirus_1a | EF535744 |
| Human_astrovirus_1e | EF535764 |
| Human_astrovirus_1b | FJ375759 |
| Human_astrovirus_1d | FJ755403 |
| Human_astrovirus_1b | FJ755404 |
| Human_astrovirus_1d | FJ823060 |
| Human_astrovirus_4c | GQ405857 |
| Human_astrovirus_6a | GQ495608 |
| Human_astrovirus_6a | GQ901902 |
| Human_astrovirus_3a | GU223905 |
| Human_astrovirus_3a | GU732187 |
| Human_astrovirus_6b | HM237363 |
| Human_astrovirus_1a | HQ398856 |
| Human_astrovirus_5c | JQ403108 |
| Human_astrovirus_2c | JQ434394 |
| Human_astrovirus_2c | JX087963 |
| Human_astrovirus_2d | JX087964 |
| Human_astrovirus_2c | KC137246 |
| Human_astrovirus_4c | KC915034 |
| Human_astrovirus_2a | KF039910 |
| Human_astrovirus_2c | KF039911 |
| Human_astrovirus_4c | KF039913 |
| Human_astrovirus_2c | KM923914 |
| Human_astrovirus_3a | KP162255 |
| Human_astrovirus_1b | KP162258 |
| Human_astrovirus_2b | L06802 |
| Human_astrovirus_2b | L13745 |
| Human_astrovirus_1a | L23513 |
| Human_astrovirus_5a | U15136 |
| Human_astrovirus_7 | Y08632 |
| Human_astrovirus_4a | Z33883 |
| Human_astrovirus_8 | Z66541 |
| Human_astrovirus_1a | NC001943 |
| Human_astrovirus_1c | AF211956 |

**Supplementary data sheet 3 |** **Deposited sequences in Genbank database**

| Sample ID | GenBank Accession Number |
| --- | --- |
| 2015S01 | MT361877 |
| 2015S02 | MT361878 |
| 2015S03 | MT361879 |
| 2015S04 | MT361880 |
| 2015S05 | MT361881 |
| 2015S06 | MT361882 |
| 2015S07 | MT361883 |
| 2015S08 | MT361884 |
| 2015S09 | MT361885 |
| 2015S10 | MT361886 |
| 2015S11 | MT361887 |
| 2015S12 | MT361888 |
| 2015S13 | MT361889 |
| 2015S14 | MT361890 |
| 2015S15 | MT361891 |
| 2015S16 | MT361892 |
| 2015S17 | MT361893 |
| 2015S18 | MT361894 |
| 2015S19 | MT361895 |
| 2015S20 | MT361896 |
| 2015S21 | MT361897 |
| 2016S02 | MT361898 |
| 2016S03 | MT361899 |
| 2016S04 | MT361900 |
| 2016S06 | MT361901 |
| 2016S07 | MT361902 |
| 2016S08 | MT361903 |
| 2016S09 | MT361904 |
| 2016S10 | MT361905 |
| 2016S11 | MT361906 |
| 2016S12 | MT361907 |
| 2016S13 | MT361908 |
| 2016S14 | MT361909 |
| 2016S15 | MT361910 |
| 2016S16 | MT361911 |
| 2016S17 | MT361912 |
| 2016S18 | MT361913 |
| 2016S19 | MT361914 |
| 2016S20 | MT361915 |
| 2016S21 | MT361916 |
| 2016S22 | MT361917 |
| 2016S23 | MT361918 |
| 2016S24 | MT361919 |
| 2016S25 | MT361920 |
| 2016S26 | MT361921 |
| 2016S27 | MT361922 |
| 2016S28 | MT361923 |
| 2016S29 | MT361924 |
| 2016S30 | MT361925 |
| 2016S31 | MT361926 |
| 2016S32 | MT361927 |
| 2016S33 | MT361928 |
| 2016S34 | MT361929 |
| 2016S35 | MT361930 |
| 2016S36 | MT361931 |
| 2016S37 | MT361932 |
| 2016S38 | MT361933 |
| 2016S39 | MT361934 |
| 2016S40 | MT361935 |
| 2016S41 | MT361936 |
| 2016S42 | MT361937 |
| 2016S43 | MT361938 |
| 2016S44 | MT361939 |
| 2016S45 | MT361940 |
| 2016S46 | MT361941 |
| 2016S47 | MT361942 |
| 2016S49 | MT361943 |
| 2016S50 | MT361944 |
| 2016S51 | MT361945 |
| 2016S52 | MT361946 |
| 2016S53 | MT361947 |
| 2016S54 | MT361948 |
| 2016S55 | MT361949 |
| 2016S56 | MT361950 |
| 2016S57 | MT361951 |
| 2016S58 | MT361952 |
| 2016S59 | MT361953 |
| 2016S60 | MT361954 |
| 2016S61 | MT361955 |
| 2016S63 | MT361956 |
| 2016S64 | MT361957 |
| 2016S65 | MT361958 |
| 2016S66 | MT361959 |
| 2016S67 | MT361960 |
| 2016S68 | MT361961 |
| 2016S70 | MT361962 |

**Supplementary Data sheet 4 | Detail of 7 infections cases**

| **Sample No.** | **Age (year)** | **Onset Date** |
| --- | --- | --- |
| 2016-1 | 7 | 18-Jan-16 |
| 2016-27 | 6 | 23-Feb-16 |
| 2016-29 | 5 | 29-Feb-16 |
| 2016-39 | 5 | 7-Mar-16 |
| 2016-64 | 12 | 12-Sep-16 |
| 2016-66 | 8 | 15-Nov-16 |
| 2015-13 | 14 | 22-Apr-15 |

**Supplementary Data Sheet 5 |Analysis of Faeces From Individuals Infected With Different HAstV genotypes**

| **Genotype** |  | **Distribution of Viral Load(genomes/g)** | | | | | | | |  |  | | | |  | |
| --- | --- | --- | --- | --- | --- | --- | --- | --- | --- | --- | --- | --- | --- | --- | --- | --- |
|  | **0** | **<10^2∽4^** | **10^4∽5^** | **10^5∽6^** | **10^6∽7^** | **10^7∽8^** | **10^8∽9^** | **10^9∽10^** | **10^10∽11^** | **10^11∽12^** | **Min genomes/g** | **Max**  **genomes/g** | **Mean SD of log genomes/g** | **Total** | |  |
| **HAstV-1** | 4 | 0 | 2 | 2 | 3 | 2 | 12 | 5 | 3 | 0 | 1.11E+04 | 9.45E+10 | 8.10±1.70 | 33 | |  |
| **HAstV-2** | 0 | 1 | 1 | 0 | 1 | 0 | 1 | 0 | 0 | 0 | 2.06E+03 | 4.54E+08 | 5.86±2.31 | 4 | |  |
| **HAstV-3** | 1 | 0 | 0 | 1 | 0 | 1 | 0 | 0 | 0 | 0 | 1.03E+05 | 1.89E+07 | 6.14±1.60 | 3 | |  |
| **HAstV-4** | 1 | 0 | 0 | 0 | 0 | 1 | 0 | 4 | 10 | 3 | 1.35E+07 | 7.53E+11 | 10.26±1.05* | 19 | |  |
| **HAstV-5** | 2 | 3 | 1 |  | 1 | 6 | 3 | 4 | 3 | 0 | 2.86E+02 | 5.59E+10 | 7.55±2.56 | 23 | |  |
| **HAstV-8** | 0 | 0 | 0 | 1 | 0 | 1 | 1 | 0 | 1 | 0 | 1.50E+05 | 3.40E+10 | 7.89±2.20 | 4 | |  |
| **untyped** | 4 | 0 | 0 | 0 | 0 | 0 | 0 | 0 | 0 | 0 | 0 | 0 | 0 | 4 | |  |
| **Total** | 12 | 4 | 4 | 4 | 5 | 11 | 17 | 13 | 17 | 3 | \ | \ | \ | 90 | |  |

HAstV-1~ HAstV-8 represent for classical human astrovirus genotype 1~8.

Differences between HAstV genotypes and the viral loads in samples were determined using analysis of variance (ANOVA).

* P<0.001

**Supplementary Data Sheet 6 | Co-Infection of Enteric Virus**

|  | **Co-Infection Rate** | **Co-Infection Sample** | **Total Positive Sample** |
| --- | --- | --- | --- |
| Norovirus Genogroup I | 31.55% | 53 | 168 |
| Norovirus Genogroup II | 9.92% | 102 | 926 |
| Sapovirus | 30.63% | 49 | 160 |
| Human Astrovirus | 28.89% | 26 | 90 |
| Enteric Adenovirus | 15.63% | 10 | 64 |
| Rotavirus A | 10.60% | 46 | 434 |
| Rotavirus B | 0.00% | 0 | 0 |
| Rotavirus C | 0.00% | 0 | 10 |

**Supplementary Data Sheet 7 | Pattern of mixed infection in HAstV-positive cases**

|  | **Pattern** | **N** |
| --- | --- | --- |
| Double Infection | NoV + HAstV | 9 |
|  | RVA+ HAstV | 3 |
|  | SaV + HAstV | 5 |
|  | HAdV + HAstV | 2 |
| Triple Infection | RVA+ SaV + HAstV | 1 |
|  | NoV+RVA+ HAstV | 1 |
|  | NoV+ SaV + HAstV | 4 |
|  | SaV + HAdV + HAstV | 1 |
| Total |  | 26 |

NoV, norovirus; HAstV, classical human astrovirus; RVA, rotavirus A; SaV. Sapovirus; HAdV, human adenovirus.

**Supplementary Data Sheet 8 |Viruses of mixed infection in HAstV-positive cases**

| **Sample No.** | **HAstV** | **Pattern of infection** | **NoVGG1 (C*q*)** | **NoVGG1 genotype** | **NoVGG2 (C*q*)** | **NoVGG2 genotype** | **SaV (C*q*)** | **SaV genotype** | **HAdV (C*q*)** | **HAdV genotype** | **RVA**  **(C*q*)** |
| --- | --- | --- | --- | --- | --- | --- | --- | --- | --- | --- | --- |
| **2016-19** | HAstV-1 | double |  |  |  |  | 35 | untyped |  |  |  |
| **2016-29** | HAstV-1 | triple |  |  |  |  | 23 | G1.1 |  |  |  |
| **2016-2** | HAstV-1 | triple |  |  | 36 | untyped |  |  |  |  |  |
| **2016-46** | HAstV-1 | triple | 36 | G1.6 |  |  | 33 | untyped |  |  |  |
| **2016-51** | HAstV-1 | triple | 36 | untyped |  |  | 37 | untyped |  |  |  |
| **2016-57** | HAstV-1 | triple | 31 | G1.4 | 25 | G2.17 |  |  |  |  |  |
| **2016-58** | HAstV-1 | triple | 33 | G1.3 | 35 | G2.17 |  |  |  |  | 36 |
| **2015-19** | HAstV-2 | double |  |  |  |  | 32 | G1.2 |  |  |  |
| **2016-31** | HAstV-2 | triple |  |  | 36 | G2.1 | 32 | G1.6 |  |  |  |
| **2016-21** | HAstV-3 | triple | 24 | G1.6 | 29 | G2.17 |  |  |  |  |  |
| **2015-5** | HAstV-4 | double |  |  |  |  | 31 | G1.2 |  |  |  |
| **2016-10** | HAstV-4 | double |  |  |  |  |  |  | 25 | type 1 |  |
| **2016-28** | HAstV-4 | double |  |  |  |  |  |  | 35 | untyped | 24 |
| **2016-34** | HAstV-4 | double |  |  | 32 | G2.17 |  |  |  |  |  |
| **2016-9** | HAstV-4 | double |  |  |  |  |  |  |  |  | 25 |
| **2016-33** | HAstV-4 | triple | 24 | G1.3 | 35 | untyped | 32 | G1.3 |  |  | 24 |
| **2015-16** | HAstV-5 | double |  |  | 18 | G2.4 |  |  |  |  | 28 |
| **2015-17** | HAstV-5 | double |  |  | 26 | G2.4 |  |  |  |  |  |
| **2016-14** | HAstV-5 | double |  |  |  |  |  |  |  |  | 36 |
| **2016-41** | HAstV-5 | double |  |  | 29 | G2.17 |  |  |  |  |  |
| **2016-42** | HAstV-5 | double |  |  | 28 | G2.21 |  |  |  |  |  |
| **2016-20** | HAstV-5 | triple | 26 | G1.5 | 20 | G2.17 |  |  |  |  |  |
| **2015-10** | HAstV-8 | double |  |  |  |  | 26 | G1.2 |  |  |  |
| **2016-32** | HAstV-8 | triple |  |  |  |  | 33 | G1.6 | 25 | type 41 |  |
| **2016-1** | untyped | double |  |  |  |  | 32 | G1.2 |  |  |  |
| **2016-5** | untyped | double |  |  |  |  |  |  |  |  | 25 |

HAstV-1~ HAstV-8 represent for classical human astrovirus genotype 1~8; NoVGG1, norovirus genogroup I; NoVGG2, norovirus genogroup II; HAstV, classical human astrovirus; RVA, rotavirus A; SaV, sapovirus; HAdV, human adenovirus. **C*q***, quantification cycle.

**Reference**

[1] Noel J S, Lee T W, Kurtz J B, et al. Typing of human astroviruses from clinical isolates by enzyme immunoassay and nucleotide sequencing[J]. J Clin Microbiol, 1995, 33(4): 797-801.

[2] Cannon J L, Barclay L, Collins N R, et al. Genetic and Epidemiologic Trends of Norovirus Outbreaks in the United States from 2013 to 2016 Demonstrated Emergence of Novel GII.4 Recombinant Viruses[J]. J Clin Microbiol, 2017, 55(7): 2208-2221.

[3] Yan H, Nguyen T A, Phan T G, et al. Development of RT-multiplex PCR assay for detection of adenovirus and group A and C rotaviruses in diarrheal fecal specimens from children in China[J]. Kansenshogaku Zasshi, 2004, 78(8): 699-709.
